# Mechanosensitive channels in lung disease

**DOI:** 10.3389/fphys.2023.1302631

**Published:** 2023-11-15

**Authors:** Mengning Zheng, Niyati A. Borkar, Yang Yao, Xianwei Ye, Elizabeth R. Vogel, Christina M. Pabelick, Y. S. Prakash

**Affiliations:** ^1^ Department of Respiratory and Critical Care Medicine, Guizhou Province People’s Hospital, Guiyang, Guizhou, China; ^2^ Department of Anesthesiology and Perioperative Medicine, Mayo Clinic, Rochester, MN, United States; ^3^ Department of Respiratory and Critical Care Medicine, The First Affiliated Hospital of Xi’an Medical University, Xi’an, Shaanxi, China; ^4^ Department of Physiology and Biomedical Engineering, Mayo Clinic, Rochester, MN, United States

**Keywords:** TRP channels, piezo channels, lung system, signal transduction, mechanosensation

## Abstract

Mechanosensitive channels (MS channels) are membrane proteins capable of responding to mechanical stress over a wide dynamic range of external mechanical stimuli. In recent years, it has been found that MS channels play an important role as “sentinels” in the process of cell sensing and response to extracellular and intracellular force signals. There is growing appreciation for mechanical activation of ion channels and their subsequent initiation of downstream signaling pathways. Members of the transient receptor potential (TRP) superfamily and Piezo channels are broadly expressed in human tissues and contribute to multiple cellular functions. Both TRP and Piezo channels are thought to play key roles in physiological homeostasis and pathophysiology of disease states including in the lung. Here, we review the current state of knowledge on the expression, regulation, and function of TRP and Piezo channels in the context of the adult lung across the age spectrum, and in lung diseases such as asthma, COPD and pulmonary fibrosis where mechanical forces likely play varied roles in the structural and functional changes characteristic of these diseases. Understanding of TRP and Piezo in the lung can provide insights into new targets for treatment of pulmonary disease.

## 1 Introduction

Transmission of mechanical forces plays an essential role in physiologic responses including organ development and baseline physiologic homeostasis, with aberrant mechanotransduction contributing to development and maintenance of disease states. Mechanosensitive channels (MS channels) are an important aspect of mechanical conduction systems first discovered in 1983 in the skeletal muscle of embryonic chicks by Falguni Guharay and Frederick Sachs (Guharay and Sachs, 1984). Since then, a range of MS channels have been found to be ubiquitous in cells from bacteria to humans. MS channels function as mechanotransducers capable of generating both electrical and ion flux downstream signals in response to external or internal stimuli. In recent years, studies have found that a variety of MS channels, including the transient receptor potential (TRP) superfamily and, more recently, Piezo channels, play important roles in disease pathogenesis including in the lung. Indeed, it is logical that MS channels are critical to lung health and disease, given the inherent mechanical nature of normal lung function, involving lifelong cyclical as well as static changes in pressure and volume related to the mechanics of breathing. Thus, understanding the roles of MS channels such as TRPs and Piezos in the lung is highly relevant. Here, we focus on TRP and Piezo channels and their roles in pulmonary disease states. We describe their structure, and systematically summarize the current research understanding on the role of these channels in the lung with the goal of providing perspective on knowledge gaps and identifying potential strategies to identify novel targets in chronic pulmonary disease.

## 2 Mechanosensitive ion channels

### 2.1 Discovery and structure of Piezo1 and Piezo2 channels

The Piezo channel family was first identified as a class of non-selective cationic mechanotransducers and are conserved from protozoa to humans (Kefauver et al., 2020). They are two subtypes of the Piezo channel family, which were first discovered by Ardem Patapoutian’s team in a mouse neuroblastoma cell line. Piezo1 and Piezo2 are encoded by Piezo1 gene (family with sequence similarity 38A, Fam38A) and Piezo2 gene (family with sequence similarity 38B, Fam38B) (Coste et al., 2010) with47% of the Piezo2 gene sequence being identical to that of Piezo1. Both Piezo1 and Piezo2 form trimeric propeller-like structures with an extracellular domain resembling three distal lobes and a central cap. These trimer structures suggest that Piezo1 and Piezo2 can use their peripheral regions as force sensors to control the central ion-conducting pore (Ge et al., 2015). The human Piezo1 and Piezo2 genes are in the 16q24.3 region of chromosome 16 and the 18p11.22-p11.21 region of chromosome 18 (Qin et al., 2021). The first report about Piezo1 and Piezo2 being present in the lung noted that lung tissues had the highest expression of these channels across organ systems (Zhao et al., 2018). Piezo1 and Piezo2 channels both exhibit rapid (i.e., ms) voltage-dependent inactivation and reversal potentials close to 0 mV (Earley et al., 2022) and are considered non-specific cation channels with substantial permeability to Ca^2+^. Piezo channels have been shown to respond to diverse types of external forces, such as compression, fluid flow, tensile forces, and even ultrasound.

Using a variety of techniques (each with unique advantages and disadvantages) such as combinations of “stretch” and “poke” with patch-clamp electrophysiology (Wu et al., 2017), there is increasing information regarding the expression and roles of Piezo channels in a variety of mechanosensitive cells and those not usually considered mechanosensitive. Recent studies have investigated the biological functions of Piezo channels in different cells. The results of these studies confirm their critical role in mechanotransduction under physiological and pathophysiological conditions (Fang et al., 2021). While the lung does express Piezo channels, the baseline or normal role of Piezo channels in the lung remains unknown. Both Piezo1 and Piezo2 should be important for lung development and in physiology. For example, Piezo1 participates in the determination of vascular structure during early development (Douguet et al., 2019). Piezo1 can regulate lung epithelial homeostasis and cell integrity by participating in cell proliferation and repair functions (Zhong et al., 2018). It may play a key role in surfactant secretion in response to alveolar type I (ATI) cell stretch, thereby triggering alveolar type II (ATII) through paracrine effects in the developing lung (Diem et al., 2020). Expression of Piezo2, derived from neural crest origin, is critical for adequate respiratory function and proper lung expansion in neonatal mice (Nonomura et al., 2017). These initial data set the stage for understanding Piezo channels in the lung.

### 2.2 Discovery and structure of TRP channels

Transient receptor potential channels (TRP) are cation channels widely involved in various life activities such as sensing of various intracellular and extracellular stimuli and maintenance of ion homeostasis (Dietrich, 2019; Dietrich et al., 2023). TRP channels were originally discovered in a so-called “transient receptor potential” mutant (TRP mutant) strain of the fruit fly *Drosophila*, lending the name. Most TRP channels consist of six membrane-spanning helices with intracellular N- and C-termini. There are 28 TRP channels in mammals classified into six subfamilies: TRPC, TRPVM, TRPVL, TRPM, TRPS and TRPN (non-mammalian NOMPC, or no mechanoreceptor potential C) (Arias-Darraz et al., 2015; Bouron et al., 2015; Lee et al., 2021). Mammalian TRP channels are activated and regulated by a variety of stimuli including mechanical stimuli, temperature, and ligand binding and are expressed in multiple organ systems. Members of the TRP channel superfamily all have similarities, such as six transmembrane segments, different degrees of sequence homology, and the ability to permeate cations, but various subfamilies demonstrate specific activation mechanisms and cation selectivity (Dietrich et al., 2023). TRP channels play a vital role in rapid perception of external stimuli by cells where they are expressed, activating downstream pathways that influence cell proliferation, differentiation, and apoptosis. Abnormal expression or activity of TRP channels are thought to contribute to human disease (Samanta et al., 2018; Dietrich, 2019). Mechanosensitivity appears to be present in nearly all TRP subfamilies, albeit to different extents: TRPV, TRPC, TRPA (ankyrin-like), TRPP (polycystin), TRPN, TRPY (yeast) and probably TRPML (mucolipins), but each subfamily has dissimilar cytoplasmic domains, indicating that these domains are not responsible for mechanosensitivity (Kung, 2005). Among TRPM channels, TRPM3, TRPM4, and TRPM7 are known to be mechanosensitive.

Unlike other ion channels, the multimodal activation characteristics of TRPs, such as activation by endogenous ligands or various environmental stimuli, confer distinct physiological and pathological roles (Yue and Xu, 2021). Most TRPs are non-selective cation channels with broad relative permeability to Ca^2+^ and Na^+^ (PCa/PNa) (Owsianik et al., 2006).Many TRP channels have been shown to be involved in a variety of physiological processes, such as regulating heat, touch, pain, odor, smell, osmotic pressure, fluid secretion, inflammation-associated changes, cell adhesion, proliferation, differentiation, migration, and apoptosis (Asghar and Tornquist, 2020). TRPC regulates multiple Ca^2+^-dependent cellular processes. TRPV4 has been reported to be present in airway epithelial smooth muscle cells and has been shown to regulate embryonic lung development, airway tone, ciliary beating frequency, and response to lung injury (Scheraga et al., 2017; Morgan et al., 2018). In a murine model of allergic asthma, mechanosensitive TRPV4 senses epithelial junction injury and initiates a rapid Ca^2+^-dependent cellular response, promoting inflammation. TRPV4 has also been shown to drive IgE-independent and mast cell-dependent bronchoconstriction. TRPV4 activation using the agonist GSK1016790A increases [Ca^2+^]_i_, releasing ATP from ASM cells (Bonvini et al., 2020; Wiesner et al., 2020). TRPV4 is expressed in ciliated epithelial cells and translates stimuli such as heat or force into Ca^2+^ signals resulting in increased ciliary beat frequency and mucociliary clearance (Lorenzo et al., 2008).

## 3 Mechanosensitive ion channels in respiratory diseases

### 3.1 Asthma

Asthma is a heterogeneous disease, usually characterized by airway hyperresponsiveness and variable airflow obstruction (Borkar et al., 2021; Borkar et al., 2022). Changes in both airway function (airway hyperresponsiveness) and structure (airway remodeling, increased airway smooth muscle proliferation, extracellular matrix deposition) are key features of asthma. While the classical focus for asthma has been on inflammation, immune mechanisms, growth factors and cell-cell interactions, more recently, MS channels are thought to play key roles in structural and functional changes associated with this disease. However, data on MS channels in asthma *per se* are relatively limited.

One study demonstrated that Piezo1 expression is higher in the bronchial epithelium of mice sensitized with ovalbumin (OVA) and aluminium hydroxide (a model of allergic asthma) compared to normal control mice sensitized with PBS. Furthermore, the same study showed that activation or increased Piezo1 leads to dysregulated tight junction (TJ) expression and impairment of small airway epithelial function (Zhou et al., 2021).

Human airway smooth muscle (HASM) and human bronchial epithelial cells (HBECs) both express multiple channels of the TRPC family, many of which play key roles in regulation of airway remodeling. TRPC6 plays an important role in regulating key functions in airway cells such as airway inflammation, migration, and chemotaxis of immune cells (Corteling et al., 2004; Gottlieb et al., 2008; Zhou et al., 2018; Chen et al., 2019; Chen et al., 2020). TRPC1 plays an important role in airway remodeling and has a significant clinical therapeutic effect on asthma. Knockdown of TRPC1 via siRNA has been found to significantly impair airway remodeling via decreasing bronchial wall thickness, smooth muscle hypertrophy and hyperplasia, decreased extracellular matrix deposition and infiltration of inflammation in mouse models of asthma sensitized with OVA, showing similar treatment effects to those seen with budesonide (Gottlieb et al., 2008; Li et al., 2019). TRPV4 has been found to play important roles in both HASM and HBECs in the setting of asthma. Increased HBEC expression of TRPV4 is associated with increased risk of fungal allergen and asthma sensitization in a mouse model of asthma (Wiesner et al., 2020). In a mouse model, TRPV4 knockout mice were found to be protected from cockroach antigen *D. Farinae*-induced airway remodeling. In HASM, TRPV4 has been found to be important in cellular response to osmotic stimuli and contributes to hypotonic airway contractility by increasing extracellular Ca^2+^, an effect independent of L-type calcium channel activation (Jia et al., 2004). The specific single nucleotide polymorphism, rs6606743 of TRPV4, was found to significantly contribute to the development of osmotic airway hyperresponsiveness in patients with asthma showing uncontrolled bronchial asthma compared to control patients having negative response to bronchoprovocation (Naumov et al., 2016). Despite these indications of mechanosensitivity, other studies suggest that TRP channels may not be inherently mechanosensitive (Gottlieb et al., 2008; Nikolaev et al., 2019), although the nuances of whether and how different TRP family members are mechanosensitive, and how they may function in specific cells of the lung undergoing mechanical forces remain to be established.

### 3.2 Pulmonary hypertension

Pulmonary hypertension (PH) is a form of high blood pressure that affects the arteries in the lungs resulting in high pressures on the right side of the heart. The pulmonary circulation is a high-flow system in which pulmonary arterial endothelial cells (PAECs), pulmonary arterial smooth muscle cells (PASMCs), and pulmonary arterial adventitial fibroblasts (PAF), are continuously exposed to mechanical stimulation, such as shear stress and pulsatility (albeit at a lower level than in the systemic circulation), and are thus more sensitive to insults and which are altered under conditions of PH (Barbeau et al., 2021).

Piezo1 channels have a key role as sensors of shear stress and determinants of vascular structure in both lung development and adult physiology. The importance of Piezo1 channels in sensing blood flow is evidenced by the dependence on Piezo1 for shear stress-evoked ionic currents, calcium influx in endothelial cells and the ability of exogenous Piezo1 to confer shear stress sensitivity in cells not responsive to stretch or stress stimuli. Interestingly, in spite of its rapid activation properties, Piezo1 activation has been found to induce proliferation of human PASMCs via mediating intracellular Ca^2+^ release. It was also shown that treatment of PASMCs with 10%FBS induced a substantial increase in proliferation rate, while knockdown of Piezo1 attenuated FBS-induced proliferation of human PASMCs compared to scrambled siRNA transfected PASMCs (Maneshi et al., 2018; Liao et al., 2021). In a chronic hypoxia model of PH in rats, immunofluorescence and patch-clamp studies demonstrated the existence of Piezo1 in freshly isolated PAECs and PASMCs (Porto Ribeiro et al., 2022). In primary isolated PAECs, Piezo1 contributed to intrapulmonary vascular relaxation by controlling endothelial [Ca^2+^]_i_ and nitric oxide (NO) production via calcium-independent pathway (Lhomme et al., 2019). Piezo1 is also upregulated in PAECs from idiopathic pulmonary arterial hypertension (iPAH) patients and in animal models of experimental PH. One proposed mechanism for pulmonary vascular remodeling that has emerged is a stretch-induced increase in phosphorylation of extracellular signal-regulated kinase (ERK) and protein kinase B, or AKT that is mediated by Piezo1 control of Ca^2+^ influx in human PAECs. Upregulation of Piezo1 has also been found to enhance [Ca^2+^]_i_ in primary isolated PASMCs from idiopathic PAH patients (Liao et al., 2021). In freshly isolated PAEC’s and PASMC’s from intrapulmonary artery rings in a rat model of chronic hypoxia-induced pulmonary hypertension, Yoda1, a Piezo1 agonist, was found to induce and activate the AKT-endothelial nitric oxide synthase (eNOS) pathway (Porto Ribeiro et al., 2022).

TRP channels have also been implicated in the pathophysiology of PH. Previous studies showed that the TRPC family plays multiple roles in regulation of [Ca^2+^]_i_ in the pulmonary artery. Inactivation of rat TRPC4 reduced acetylcholine induced Ca^2+^ increases in PASMCs and resulted in reduction of the severity of pulmonary arteriopathy obliterans in a Sugen 5,416/hypoxia/normoxia rat model of PAH (Alzoubi et al., 2013). TRPC6 is highly expressed in PASMCs in patients with idiopathic pulmonary arterial hypertension (iPAH) and is involved in PASMC proliferation by initiating and promoting cell cycle growth, and by triggering gene transcription via transcription factor phosphorylation (Yu et al., 2004).

A study showed (Daneva et al., 2021) that TRPV4 channels in endothelial cell caveolae maintain a low pulmonary arterial pressure under normal conditions. Moreover, endothelial caveolin-1–TRPV4 channel signaling lowers pulmonary arterial pressure, and impairment of endothelial caveolin-1–TRPV4 channel signaling contributes to elevated pulmonary arterial pressure in PH through protein kinase C signaling pathway. Furthermore, chronically hypoxic, and monocrotaline-exposed rats showed elevated levels of TRPV4 protein expression in animal models of PH. Morphometric analysis indicated that adventitial remodeling is reduced in PH-induced TRPV4−/− mice (Cussac et al., 2020). CAV1 and TRPV4 therefore may play a pathophysiological role in PAH (Perez-Vizcaino et al., 2021).

### 3.3 Acute respiratory distress syndrome (ARDS)/Acute lung injury (ALI)

Mechanical ventilation is a critical intervention for many patients with respiratory failure due to ARDS/ALI. However, paradoxically, mechanical ventilation may also produce excessive mechanical stress that can directly exacerbate lung injury, a syndrome known as ventilator-induced lung injury (VILI). The pathobiology of VILI and ARDS has many inflammatory features, including increased pulmonary vascular permeability due to loss of endothelial cell barrier integrity, leading to alveolar flooding (Wang et al., 2017). Excessive mechanical stretch during ARDS is thought to induce activation of Piezo1 and its downstream target calpain via Ca^2+^ influx. This leads to disassembly of endothelial adherens junctions (AJs) and further promotes lung endothelial barrier breakdown and vascular hyperpermeability (Jiang et al., 2021). Autoregulation of Piezo1 signaling in the endothelium is a key factor in the pathogenesis of VILI. Thus, increasing Piezo1 expression or pharmacologically activating Piezo1 signaling may be an effective therapeutic strategy for treating or preventing VILI (Zhong et al., 2020).

In mice exposed to mechanical ventilation, the pulmonary vascular permeability of Piezo1iEC^−/−^ mice was significantly upregulated compared with that of Piezo1fl/fl mice, and Piezo1fl/fl mice demonstrated significant upregulation in lung vascular permeability compared to vehicle Piezo1fl/fl mice when stimulated with high-volume mechanical ventilation. This implies that downregulation of endothelial Piezo1 signaling may be a crucial factor in the pathogenesis of VILI (Zhong et al., 2020). Another study showed that stretch-induced Ca^2+^-signals depended on Ca^2+^-entry via Piezo1 channels, which regulate stretch-induced surfactant secretion from ATII cells (Diem et al., 2020). Also, in ECs, Piezo1 activation by elevated pulmonary microvascular pressure has been found to mediate capillary stress failure, leading to lung edema (Friedrich et al., 2019).

Studies in various forms of lung injury animal models have also provided proof that TRPV4 channels play crucial roles in disruption of the alveolar-capillary barrier, subsequently causing lung injury (Bihari et al., 2017). Another study also implicated TRPV4 channels from capillary endothelial cells, alveolar epithelial cells, and immune cells in the pathogenesis of lung injury (Sonkusare and Laubach, 2022). TRPV4 activation using specific TRPV4 agonist, GSK-1016790A induces epithelial and capillary endothelial swelling, blebbing and shedding, leading to alveolar flooding (Villalta et al., 2014). The orally active TRPV4 inhibitor GSK2193874 reduces Ca^2+^ flux and attenuates pulmonary edema in cultured cells in response to increases in venous pressure that occur during heart failure (Thorneloe et al., 2012). TRPV4 inhibitors also attenuated VILI-associated macrophage activation (Thorneloe et al., 2012). Furthermore, TRPV4 inhibitors reduce pulmonary edema in isolated perfused mouse lungs (Hamanaka et al., 2010). Therefore, it may be a potential therapeutic target in the pathogenesis of ALI/ARDS.

### 3.4 Lung fibrosis

Idiopathic pulmonary fibrosis (IPF) is a chronic fibrotic interstitial lung disease (ILD) of unknown etiology characterized radiologically and histopathologically by usual interstitial pneumonia (UIP), with abnormal behavior of alveolar epithelial cells and migration, proliferation, and activation of mesenchymal cells, leading to fibroblast-myofibroblast transition (FMT). Activated myofibroblasts secrete large amounts of extracellular matrix molecules leading to subsequent fibrosis and destruction of normal lung architecture (Tzilas et al., 2022).

TRPV4 has been found to play a role in regulating pulmonary fibrosis. One study showed that TRPV4 activity is increased in IPF patient-derived lung fibroblasts (Rahaman et al., 2014). Expression of TRPV4 has also been found to be increased in bronchiolar and alveolar epithelium of IPF patients. In another study, TRPV4 KO mice were protected from bleomycin-induced pulmonary fibrosis. Inhibition of TRPV4 channel activity has been found to abrogate the molecular signaling that is essential for myofibroblast differentiation, such as with TGFβ signaling (Rahaman et al., 2014). The anti-fibrotic effect of the drug pirfenidone is partially mediated by TRPV4, and endogenous ligands of TRPV4 in bronchoalveolar lavage fluid may be a biomarker to distinguish pirfenidone responders (Kuronuma et al., 2022).

TGF-β-activated epithelial-mesenchymal transition (EMT) is a major process involved in the pathogenesis of pulmonary fibrosis. Activation of Piezo1 by 12-Gy irradiation (a model for fibrosis) can upregulate the expression of TGF-β1 and induce EMT through Ca^2+^/HIF-1α signaling in RLE-6TN cells (Huang et al., 2021). Piezo1-mediated ATP release, driven by mechanical stretch, also enhances lung fibrosis and EMT in human lung epithelial cells *in vitro* and in a two-hit model of mechanical ventilation after acid aspiration-induced lung injury in mice *in vivo*. Moreover, the same study demonstrated genetic deletion or pharmacological blockade of Piezo1 in lung epithelial cells has been found to prevent increased EMT and enhanced fibrosis by mechanical ventilation or stretch (Fang et al., 2022).

Regarding treatment option for IPF, preventing lung fibroblasts from sensing the stiffness of their environment and responding to it by using sensor or transcription factor inhibitors might have more potential than blocking cross-linking enzymes relevant to fibrosis. For example, targeting the sensing of altered Piezo channels or the transcription factors that direct gene expression in response to stiffness is expected to downregulate multiple important mechanotransduction pathways such as matrix (TG2, LOX) sensing, Wnt signaling and metabolism pathways (Freeberg et al., 2021). Thus, Piezo channel expression and function might be of substantial importance in fibrosis.

### 3.5 Chronic obstructive pulmonary disease (COPD)

COPD is a heterogeneous lung condition characterized by chronic respiratory symptoms due to abnormalities of the airways (bronchitis, bronchiolitis) and/or alveoli (emphysema) that cause persistent, often progressive, airflow obstruction. TRPV4 has multiple functions in regulating COPD-associated airway physiology. Gene polymorphisms have been found to increase TRPV4 transcript levels associated with COPD (Zhu et al., 2009). Expression of TRPV4 has been found in human airway smooth muscle cells, where it acts as an osmolarity sensor in the airway (Jia et al., 2004). Similarly, in a human bronchial epithelial (HBE) cell line, the channel TRPV4 senses volume changes and may provide a pathway for Ca^2+^ influx in hypotonic solutions, thereby activating maximal K^+^ channels (Fernandez-Fernandez et al., 2002). Insufficient TRPV4 activation due to airway epithelial hypotonia in ΔF508 mutants may be associated with bronchial hypersecretion, a feature of COPD (Joos et al., 2002). TRPV4 has also been more strongly associated with COPD status as a binary variable than with a quantitative measure of airflow obstruction in COPD cases, implying that genetic variants in TRPV4 may influence COPD susceptibility (Zhu et al., 2009).

### 3.6 Obstructive sleep apnea/hypopnea syndrome (OSAHS)

OSAHS is recurrent apnea due to narrowing or obstruction of the upper airway during sleep, resulting in a series of symptoms such as hypoxia and arrhythmia, and even sudden death due to suffocation. Piezo2 can act as an airway stretch sensor, and Piezo2-mediated mechanotransduction within various airway-innervating sensory neurons could be important for establishing efficient respiration at birth and maintaining normal respiration in adults. Optogenetic activation of Piezo2-positive vagal sensory neurons causes apnea in adult mice (Nonomura et al., 2017). OSAHS causes decreased intracranial blood oxygen concentration and damages hippocampal neurons. Piezo2 expressed in the hippocampus may help explain relationships between the brain and the respiratory system (Wang and Hamill, 2021).

## 4 Discussion

Increasing evidence suggests that MS channels play a key role in the pathogenesis of lung disease. MS channels are not only activated by mechanical stretch/shear stress but are also regulated by other external/internal stimuli that alter lung structure and function. MS channels play a key role in multiple lung diseases as summarized in Table 1. In the past decade, identification of new families of mechanosensitive ion channels such as Piezo channels and astonishing insights into the studies of MS channels have driven further development in the arena of mechanotransduction. While there is some emerging information on the roles of MS channels (Piezo and TRP) in the lung, not much is known regarding their differential existence and function in normal homeostasis and pulmonary pathologies. Therefore, further investigations are needed to elucidate how these channels regulate downstream effects that result in chronic lung disease.

**TABLE 1:** Effects of mechanosensitive ion channels in lung cells towards health and disease.

Disease	Channels	Cell types	Mechanism
Pulmonary Hypertension	Piezo1	Pulmonary arterial smooth muscle cells	Cell proliferation (Maneshi et al., 2018; Liao et al., 2021)
		Endothelial cells	Intrapulmonary vascular relaxation via [Ca^2+^]_i_ and NO production (Lhomme et al., 2019)
		Pulmonary arterial endothelial cells	Phosphorylation of ERK and AKT by Ca^2+^ influx (Liao et al., 2021)
		Pulmonary arterial smooth muscle cells	Increased [Ca^2+^]_i_ (Liao et al., 2021)
	TRPC4	Pulmonary arterial smooth muscle cells	Reduced store operated Ca^2+^ entry (Alzoubi et al., 2013)
		Vascular Endothelial Cells	Redox-sensitive Ca^2+^-permeable channel (Alzoubi et al., 2013)
	TRPC6	Pulmonary arterial smooth muscle cells	Cell proliferation (Yu et al., 2004)
	TRPV4	Pulmonary arterial smooth muscle cells	Cell proliferation, migration, and cytoskeletal reorganization (Daneva et al., 2021)
		Endothelial cells	Maintain low pulmonary arterial pressure under normal conditions, cell proliferation and migration (Perez-Vizcaino et al., 2021)
		Pulmonary arterial fibroblasts	Adventitial remodeling (Cussac et al., 2020)
Asthma	Piezo1	Small airway epithelial cells	Airway epithelial function and disordered tight junction protein expression via increased [Ca^2+^]_i_ (Zhou et al., 2021)
	TRPC1	Asthma models	Airway remodeling (Gottlieb et al., 2008; Li et al., 2019)
	TRPV4	Bronchiolar epithelial cells	Fungal allergen and asthma sensitization (Wiesner et al., 2020)
		Human airway smooth muscle cells	Airway contractility and hyperresponsiveness (Jia et al., 2004)
ARDS/ALI	Piezo1	Endothelial cells	Capillary stress failure, promote lung endothelial barrier breakdown and vascular hyperpermeability in pathogenesis of ventilator-induced lung injury (Friedrich et al., 2019; Zhong et al., 2020)
		Alveolar type II cells	Regulate stretch-induced surfactant secretion (Diem et al., 2020)
	TRPV4	Epithelial, capillary endothelial cells	Cell swelling, blebbing and shedding (Villalta et al., 2014)
		Capillary endothelial cells, alveolar epithelial cells, and immune cells	Lung injury (Sonkusare and Laubach, 2022)
Lung Fibrosis	TRPV4	Lung fibroblast	Promote myofibroblast differentiation (Rahaman et al., 2014)
	Piezo1	Epithelial cells	Promote epithelial-mesenchymal transition, enhance fibrosis by mechanical ventilation or stretch (Huang et al., 2021; Fang et al., 2022)
COPD	TRPV4	Airway epithelium	Ca^2+^ entry to trigger bronchial hypersecretion (Joos et al., 2002)
		Airway smooth muscle cells	Osmolarity sensor (Jia et al., 2004)
		Bronchial epithelial cells	Ca^2+^ influx (Fernandez-Fernandez et al., 2002)
OSAHS	Piezo2	Hippocampal neurons	Decrease intracranial blood oxygen concentration and impair neuronal responses (Wang and Hamill, 2021)
		Nucleus ambiguus	Airway stretch sensor, maintaining normal respiration in adults (Nonomura et al., 2017)

Mechanosensing of the environment is a crucial aspect of cell fate and is required by organisms to receive mechanical perturbations and convert them into electrochemical signals (Santoni et al., 2021). MS channels are thought to be most important in modulating mechanosensory or mechanotransduction processes in numerous cell types and organ systems (Wal et al., 2020). Over the past few decades, multiple types of channels have been recognized as *bona fide* MS channels, and dysfunction or dysregulation of these MS channels has been implicated in various pathological conditions and diseases. Cells in the lung parenchyma, airways, and pulmonary and bronchial vasculature are constantly exposed to various mechanical forces (e.g., shear stress, stretch, osmotic pressure, heat, pain andhydrostatic pressure) associated with lung expansion, perfusion, and strenuous physical activity (Nonomura et al., 2017; Li et al., 2018). Because various MS channels are expressed within the same cell types in lung tissues, studies are needed to establish exactly how these cells integrate different mechanical and physiological outputs during disease conditions of the lungs. Here, it will be important to understand the inherent mechanosensitivity of TRP channels *per se* as opposed to mechanosensitivity conferred on TRP channels as a result of Piezo channels, i.e., indirect effects. Interactions may also occur at the intracellular levels. For example, TRP proteins are integral to agonist and store depletion activated Ca^2+^ entry channels that are in turn regulated directly by sarcoendoplasmic reticulum (SR) Ca^2+^ release channels (Sienaert et al., 1996; Boulay et al., 1999). Piezo1 can modulate SR Ca^2+^ release dynamics via such channels (Santana Nunez et al., 2023), and thus the SR may be a point of interaction. Piezo1 transduces increased myocardial forces into chemical signals that initiate hypertrophic signaling via a close physical interaction with TRPM4 (Yu et al., 2022). Whether such chemically mediated interactions between TRP and Piezo channels are involved in the lung remain to be explored.

**FIGURE 1:**
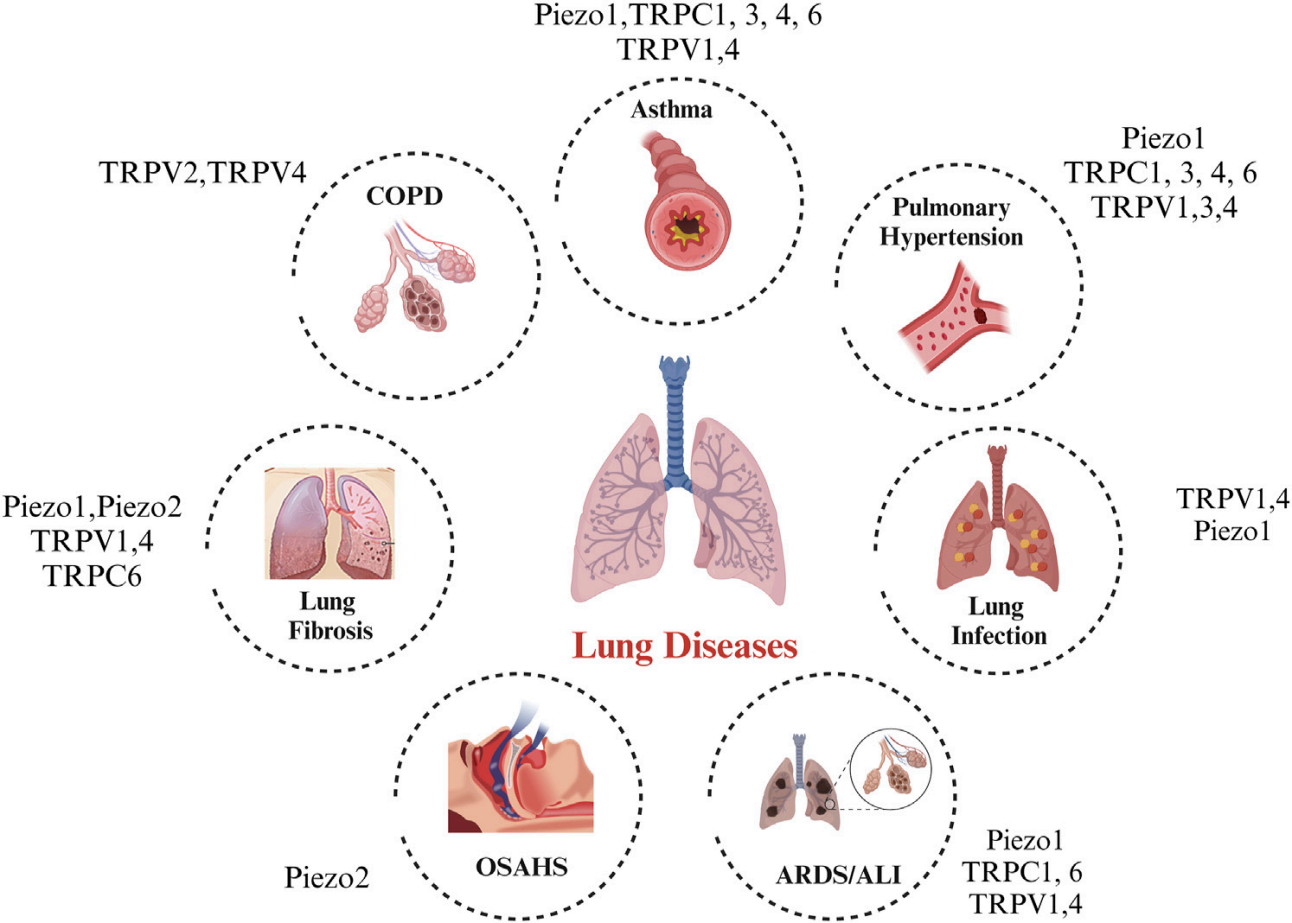
Legend: A landscape of the *in vitro* and *in vivo* roles of mechanosensitive ion channels in lung disease.

With our present understanding of the link between MS channels and lung resident cells, there remain several gaps in knowledge left to be filled, such as: 1) can MS channels be mutually regulated and be involved in stimulating pathological conditions? 2) in addition to the MS channels implicated in this review, are there other newer mechanosensitive candidates that may play a role in lung health and disease 3) while most of the studies performed are using *in vitro* cultured cells, a need to corroborate these findings *in vivo* is crucial and should be considered. Figure 1.
